# Estimation of Genetic Variance and Breeding Values for Infectious Disease Susceptibility From Simulated Longitudinal Data Using Generalized Linear Mixed Models Based on Transmission Dynamics

**DOI:** 10.1111/jbg.70049

**Published:** 2026-04-09

**Authors:** A. D. Hulst, R. Pong‐Wong, A. Doeschl‐Wilson, M. C. M. De Jong, P. Bijma

**Affiliations:** ^1^ Infectious Disease Epidemiology Wageningen University & Research Wageningen the Netherlands; ^2^ Animal Breeding and Genomics Wageningen University & Research Wageningen the Netherlands; ^3^ The Roslin Institute University of Edinburgh Midlothian UK

## Abstract

Recent theoretical work shows that the potential of genetic selection to reduce the prevalence of infectious diseases is much larger than expected from classical quantitative genetic theory, due to indirect genetic effects that arise in the transmission process. However, to fully benefit from these indirect effects, we need to estimate genetic parameters and breeding values, which requires statistical methods tailored to the transmission process. Here, we evaluate Generalized‐Linear‐Mixed Models (GLMMs) implemented using software commonly used in animal breeding to estimate genetic parameters and breeding values for susceptibility of hosts to infection, using simulated data of epidemics. Longitudinal records of individuals' infection state provide information on the order of infection, as well as on the exposure dose of non‐infected animals. Such information can be harnessed to estimate genetic parameters for susceptibility, and can be included in a GLMM as a so‐called offset. Therefore, we used longitudinal records of individual infection state to assess the impact of sampling interval, population structure, infection characteristics, and model formulation on the estimated genetic variance and breeding values for susceptibility. The results show that a GLMM fitted to longitudinal records of individual binary infection state can produce accurate and unbiased estimates of genetic variance, as well as good prediction accuracies of breeding values for susceptibility to an infectious disease. Of the data requirements, the time interval between consecutive observations on individual infection state was the main factor affecting estimation, while group size had a limited effect. The required observation interval depends on the infection and recovery rates of individuals. The GLMM thus seems an accurate and easily implementable model to estimate genetic parameters and breeding values for susceptibility when dense longitudinal records on individual infection status are available.

## 1 Introduction

Genetic selection of animals against infectious diseases, as an addition or alternative to other control measures, has attracted interest for several decades (e.g., Bishop et al. 2010). The common approach in animal breeding is to measure infectious disease resistance as the binary infection state of the animal (healthy/infected = 0/1), and analyse these data either with a regular linear model or a so‐called threshold model (e.g., Pérez‐Cabal et al. 2009; Welderufael et al. 2017). While linear models treat the binary observation as a continuous Normally‐distributed variable, threshold models use a GLMM‐like approach to link the binary observation to an underlying normally distributed liability (Dempster and Lerner 1950; Gianola 1982; De Villemereuil et al. 2016). Importantly, both approaches do not incorporate the transmission dynamics of infections, such as variation in exposure of individuals to infected herd mates. Moreover, estimated genetic parameters and breeding values from these two approaches do not have a clear epidemiological interpretation, and are, therefore, not predictive of response to selection (Bijma et al. 2022). This discrepancy is illustrated by unexpectedly large responses to selection found in models for infectious diseases, such as for gastro‐intestinal parasite infections, where responses clearly deviated from predictions based on classical quantitative genetic models (Bishop and Stear 1997, 1999, 2003).

Recent theoretical work, however, shows that the transmission dynamics of infections cause indirect genetic effects, resulting in a much larger potential of selection for lower infectious disease prevalence than expected from classical quantitative genetic models (Hulst et al. 2021; Bijma et al. 2022). Indirect genetic effects are effects of an individual's genes on trait values of other individuals. For example, individuals who are less susceptible to becoming infected also infect fewer others, simply because they are less likely to be infected themselves.

Epidemiological models are typically expressed in terms of susceptibility, whereas breeders often focus on disease resistance. Disease resistance in the animal breeding context is often defined in terms of the phenotype measured and can refer to various host response mechanisms (e.g., resistance to becoming infected, resistance to developing disease symptoms when exposed to infectious pathogens, or host ability to inhibit within‐host pathogen replication; Doeschl‐Wilson et al. 2021). However, susceptibility to infection/disease is merely the opposite of resistance to infection/disease; individuals with a high susceptibility have a low resistance, and vice versa.

A more important difference between classical quantitative genetics of threshold characters and genetic epidemiology lies in the relationship of genetic variation in host response to infection with disease prevalence. Classical quantitative genetic models, such as the threshold model, suggest that genetic reduction of infection prevalence becomes increasingly difficult at lower prevalence because heritability on the observed binary scale vanishes when the prevalence approaches zero (Robertson 1950). In full contrast, genetic epidemiological models show that the genetic variation in the prevalence of an infection increases strongly when prevalence goes down, allowing in principle for eradicating a disease by means of genetic selection (Hulst et al. 2021; Bijma et al. 2022).

Since one of the host traits affecting the transmission process is susceptibility to infection (Anche et al. 2014; Anacleto et al. 2015), accurate estimates of genetic variation and breeding values for susceptibility are needed for correct prediction of response to selection in infection prevalence. Statistical models tailored to the transmission process are expected to be superior to classical threshold models because the infection state of an individual depends on its exposure to infectious material, for example, from infected herd mates, which will fluctuate over time (Hulst et al. 2021). The use of fixed effects in the model, such as a herd‐year‐season effect, will not properly account for such variation in exposure because part of the exposure has a genetic background, and because herd‐year‐season classes typically cover a time period that is too long to capture the fine‐grained temporal fluctuation in exposure. Moreover, the binary infection records of individuals provide information on the exposure of herd mates, which can be utilised. For example, when longitudinal data on the infection state of herd members is available, then the longitudinal variation in exposure is in principle known and does not need to be estimated but can inform the model. Therefore, for accurate estimation of genetic variance and breeding values for susceptibility, we need models that account for the exposure of susceptible individuals to infectious herd mates.

Generalized‐Linear‐Mixed‐Models (GLMMs) that are founded in epidemiological theory and which were developed with the aim to estimate transmission parameters are able to estimate genetic parameters and individual genetic effects for susceptibility (Velthuis et al. 2003; Lipschutz‐Powell et al. 2012; Anche et al. 2015; Biemans et al. 2019). Results of Biemans et al. (2019) suggest that the GLMM can relatively accurately estimate breeding values for susceptibility, even with a limited amount of data. GLMMs are available in standard animal‐breeding software (e.g., ASREML; Gilmour et al. 2015) and require relatively limited computation time, which is relevant for practical implementation. However, the performance of the GLMM for estimation of genetic parameters for susceptibility has never been properly validated.

Here we evaluate how precision of variance estimates and accuracies of EBV for susceptibility, estimated with a GLMM, depend on the genetic variance in susceptibility, population structure, available data, and model formulation. We also compare results of the GLMM to those of an LMM. We use longitudinal data from simulated epidemics and the GLMM implemented into ASREML software. We investigate the effects of the size of the genetic variance in susceptibility, and the transmissibility of the infection ($R_{0}$) on the precision of genetic parameter estimates and breeding value accuracy for susceptibility. With respect to data requirements, we focus on observation interval and contact‐group size. The results show how different types of disease data and trial designs impact the precision of genetic variance estimates and accuracy of EBVs for host susceptibility.

## 2 Methods

**FIGURE 1:**
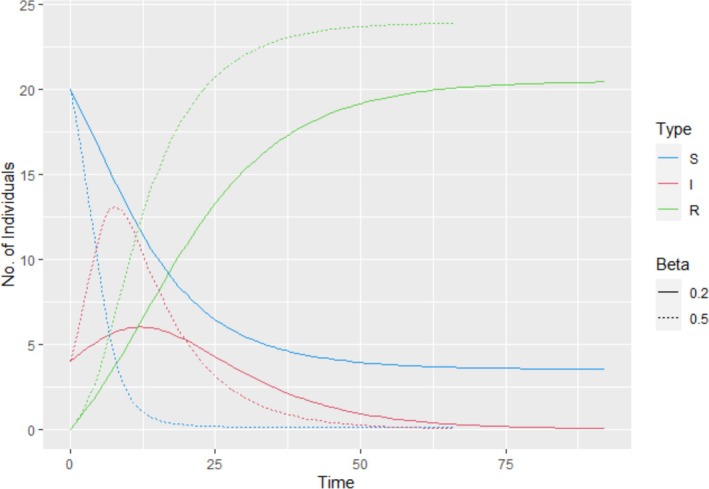
Typical epidemic curves for a contact group of 24 individuals with transmission rate parameter (β) of 0.2 or 0.5, and recovery rate parameter (α) of 0.1 (such that $R_{0}=\beta /\alpha$ = 2 or 5). Dying out of the epidemic is represented by stopping of the line. [Colour figure can be viewed at wileyonlinelibrary.com]

To be able to evaluate the genetic parameter estimates for host susceptibility obtained from GLMMs, we simulated epidemics of an infection using a Susceptible‐Infectious‐Recovered (SIR) compartmental epidemiological model. In an SIR‐model, animals can be in one of three states; Susceptible (i.e., they can become infected), Infected, or Recovered. Infected animals are assumed to be immediately infectious, and recovered animals represent animals that are immune and can thus not be infected again. Figure 1 illustrates the typical evolution of the frequency of infection states in the population over time for the SIR model.

We simulated a population consisting of contact groups within which the infection can be transmitted. In each contact group, a few random individuals were selected as seeders to start the epidemic. The infection state of each individual (*S*, *I* or *R*) was checked at regular intervals, mimicking a real‐life setup where exact infection and recovery times are usually unknown, but individual infection state can be sampled at regular intervals. The resulting longitudinal data on individual infection state was then used as input for the estimation of genetic parameters and breeding values.

To assess the performance of the GLMM and data requirements, we simulated different scenarios with different levels of genetic variance in susceptibility, average transmissibility of the infection ($R_{0}$), group size, and sampling interval. Furthermore, we investigated the effect of including different model terms (e.g., a fixed group effect to account for differences in exposure between groups) in the GLMM. Lastly, we compared the output of the GLMM to results obtained with the Linear Mixed Model and threshold model typically used in animal breeding.

### 2.1 Data Simulation Using an Epidemiological Model

Epidemics were simulated using a stochastic version of the SIR model in R (R Core Team 2020), where individuals only differed in their susceptibility to infection. A Doob‐Gillespie algorithm was used to simulate the epidemics as a series of stochastic events (i.e., individuals' infection and recovery) that follow a Poisson process, as described, for example, in Pooley et al. (2020). Briefly, the Doob‐Gillespie algorithm represents stochasticity in Markovian compartmental (e.g., SIR) models (i.e., models for which the transition rates depend solely on the current state of the system).

For a susceptible individual *i*, the infection event, that is, the transition from the susceptible (*S*) to the infectious (*I*) state, happens at a stochastic rate

(1) $$S_{i}\to I:\beta \gamma_{i}\frac{I}{N}$$

where β denotes the average transmission rate parameter, $\gamma_{i}$ the relative susceptibility of individual *i*, and $\frac{I}{N}$ the proportion of infectious individuals in the contact group, reflecting the exposure of *i* to infectious group mates.

The second event, recovery, is the transition of an individual from infectious (*I*) to recovered (*R*). In our simulations, the recovery period was gamma‐distributed with shape‐parameter 3 and rate 0.3, resulting in an average duration of the infectious period of 10 days (with a corresponding recovery rate of α=0.1). A gamma‐distributed infectious period is more biologically realistic than the conventional exponential distribution (Pooley et al. 2020). We did not consider genetic variation in the recovery period.

Individual susceptibility (γ) was simulated following Hulst et al. (2021), that is, assuming an infinitesimal genetic model and a lognormal distribution,

(2) $$\begin{array}{c} \gamma_{i}=e^{A_{\log_{\gamma },i}+E_{\log_{\gamma },i}} \end{array}$$

where $A_{\log_{\gamma },i}$ denotes the additive genetic part of individual log‐susceptibility, and $E_{\log_{\gamma },i}$ the permanent environmental part of individual log‐susceptibility. Both $A_{\log_{\gamma }}$ and $E_{\log_{\gamma }}$ were normally distributed, with mean zero and variance $a_{\log_{\gamma }}^{2}\sigma_{P_{\log_{\gamma }}}^{2}$ for the genetic part, and $\left(1-a_{\log_{\gamma }}^{2}\right)\sigma_{P_{\log_{\gamma }}}^{2}$ for the permanent environmental part. Here $\sigma_{P_{\log_{\gamma }}}^{2}$ denotes the total ‘phenotypic’ variance in the logarithm of susceptibility, and $a_{\log_{\gamma }}^{2}$ the additive genetic proportion of the full permanent variance in the logarithm of susceptibility (i.e., on the underlying normal scale; Hulst et al. 2021). We refer to $\sigma_{P_{\log_{\gamma }}}^{2}$ as a phenotypic variance, because it is the sum of a genetic and environmental part, even though phenotypic values for susceptibility are not directly observable. Thus, $A_{\log_{\gamma }}$ is the breeding value, as common in quantitative genetics, for the logarithm of susceptibility, and γ is lognormally distributed. One of the advantages of the lognormal distribution of γ is that the transmission rate will never become negative (which is not possible). The permanent environmental part of individual susceptibility, $E_{\log_{\gamma }}$, was included in the simulation, because repeated observations on the same individuals will be used in the genetic analysis.

Furthermore, for convenience, we want the mean of γ to be 1, such that our parameter β in Equation (1) is the actual mean transmission rate parameter. However, the mean of the lognormal distribution equals $e^{\mu +\frac{1}{2}\sigma^{2}}$, where μ is the mean and $\sigma^{2}$ the variance on the normally distributed scale. In quantitative genetics, *A* and *E* are typically defined with a mean of zero. However, since $e^{\frac{1}{2}\sigma^{2}}>0$, the mean susceptibility (γ) will be greater than one when $\mu =\bar{A_{\log_{\gamma }}}+\bar{E_{\log_{\gamma }}}=0$ while the variance of *A* + *E* is larger than zero. For a small variance of *A* + *E* the deviation from 1 is small, but for large variances the deviation becomes quite problematic for the comparison of scenarios (e.g., for $\sigma_{P_{\log_{\gamma }}}^{2}=1$ the mean of the lognormal is 1.65). Thus, for each simulation we divided β by the mean of the lognormal distribution given the simulated phenotypic variance in the logarithm of γ (i.e., by $e^{\mu +\frac{1}{2}\sigma_{P_{\log_{\gamma }}}^{2}}$), such that the transmission rate was corrected for the effect of simulated variance, resulting in a mean transmission rate parameter equal to *β*.

### 2.2 Population Structure

The population was simulated with a full‐sib half‐sib structure, where 96 unrelated sires were mated to 5 unrelated dams each, producing 10 offspring per mating, resulting in a total offspring population of 4800 animals. Offspring breeding values for log‐susceptibility ($A_{\log_{\gamma }}$) were simulated assuming Mendelian inheritance and the infinitesimal model (Fisher 1918). These offspring were randomly allocated to groups of size *N*. Epidemics were simulated within groups, starting with infection of four randomly selected seeder individuals per group, and no transmission between groups. At fixed timepoints with interval Δt, the infection state of each individual was recorded. Since the epidemics in the contact groups had a limited time span because at some point there are no infected individuals left (Figure 1), the number of time points recorded was not the same in all scenarios, but it depended on the duration of the epidemic (*T*) and the sampling interval (Δt). Thus, the total number of observed timepoints per scenario was $\frac{T}{\Delta t}$. Hence, at the same *T*, scenarios with smaller Δt had more observations than scenarios with larger Δt.

### 2.3 Statistical Analysis With GLMM

We used a Generalized‐Linear‐Mixed‐Model tailored to infectious disease data, following Velthuis et al. (2003), Lipschutz‐Powell et al. (2014), Anche et al. (2015), and Biemans et al. (2019). The GLMM models the probability, $P_{i}\left(t\right)$, of a susceptible individual, *i*, to become infected in the time interval Δt,

(3) $$P_{i}\left(t\right)=1-e^{-\beta \gamma_{i}\frac{I}{N}\Delta t}$$

where $e^{-\beta \gamma_{i}\frac{I}{N}\Delta t}$ is the zero term of the Poisson distribution, representing the probability for *i* to escape infection in the interval Δt. The term in the exponent is the negative of the mean number of infections of *i* during Δt, which is the product of the transmission rate parameter β, the susceptibility $\gamma_{i}$ of *i*, the fraction of infected group mates at the start of the interval $\frac{I}{N}$, and the length of the interval (Δt).

Whether or not an individual becomes infected in an interval, indicated by *y* = 0/1, follows a binomial distribution, with binomial total 1 and probability $P_{i}\left(t\right)$,

$$y_{i}\sim \mathrm{Bin}\left(1,P_{i}\left(t\right)\right).$$

For binary infection data resulting from a Poisson process, as simulated above, the appropriate link function to connect the expected value of *y* to the explanatory variables is the complementary log–log function (cloglog; Velthuis et al. 2003). A GLMM tailored to longitudinal records of individual binary infection state, therefore, follows from applying the cloglog transformation to Equation (3), giving:

(4) $$\begin{array}{c} \text{cloglog}\left(P_{i}\left(t\right)\right)=\log\left(\beta \right)+\log\left(\gamma_{i}\right)+\log\left(\frac{I\left(t\right)}{N}\Delta t\right) \end{array}$$

where $P_{i}\left(t\right)$ is the probability of infection of susceptible individual *i* during an interval of duration ∆t, $\log\left(\beta \right)$ is the intercept, $\log\left(\gamma_{i}\right)$ the logarithm of the susceptibility of individual *i*, and $\log\left(\frac{I\left(t\right)}{N}\Delta t\right)$ an offset, that is, an explanatory variable with a known value, separately for each group and each interval. The offset accounts for the known exposure of individual *i* to infectious group mates, which depends on the fraction of group mates that are infectious at that time, $I\left(t\right)/N$, and the duration of the sampling interval (Δt). For large Δt, the offset is an approximation because $I\left(t\right)$ may change during the interval, which we did not account for. Thus, the input data for the GLMM has a row for each individual that is susceptible at the beginning of an interval, and contains a column indicating whether (*y* = 1) or not (*y* = 0) that susceptible individual got infected in that interval (whether it is a case), and a column containing $\log\left(\frac{I\left(t\right)}{N}∆t\right)$ accounting for the exposure of the individual to infectious group mates at the start of the interval.

Note that *y* is not the infection state of an individual, but denotes whether or not a susceptible individual got infected during the interval, that is, whether its state changed from *S* to *I* within the interval. Thus, the dataset only includes individuals that were in state *S* at the start of the interval. For example, for a total of six observation intervals and an individual who becomes infected in the fourth interval, the *y*‐values would be as follows: 0 0 0 1 NA NA (NA indicating missing). Thus each interval before infection yielded an observation, with a corresponding offset.

To determine the effect of different model terms on the estimation of genetic variance and breeding values for susceptibility, we fitted four different GLMMs to the simulated data, using ASREML (Gilmour et al. 2015). Table 1 gives an overview of the models. Model 1 can be considered as the ‘full model’:

$$\begin{array}{c} \text{cloglog}\left(P_{\mathrm{ikt}}\right)=c_{0}+{\text{Group}}_{k}+\mathrm{Tim}e_{t}+\text{Grou}p_{k}.\mathrm{Tim}e_{t}+\\ A_{\log_{\gamma },i}+E_{\log_{\gamma },i}+\log\left(\frac{I_{\mathrm{kt}}}{N}\Delta t\right)\;\left(\text{model}\;1\right) \end{array}$$

where $c_{0}$ is the intercept, which will give the estimate for $\log\left(\beta \right)$, Group_*k*_ and $\mathrm{Tim}e_{t}$ are fixed effects for group and observation period, $\text{Grou}p_{k}.\mathrm{Tim}e_{t}$ is a random interaction term between group and observation period, $A_{\log_{\gamma },i}$ is the random genetic effect for log‐susceptibility of individual *i*, $E_{\log_{\gamma },i}$ the random permanent effect for log‐susceptibility of individual *i*, and $\log\left(\frac{I_{\mathrm{kt}}}{N}\Delta t\right)$ an offset for observation period *t* in group *k*. In model 1, the logarithm of susceptibility $\log\left(\gamma \right)$ (Equation 4) is separated into a random genetic effect with underlying pedigree relationship matrix, and a random permanent environmental effect, with $\log\left(\gamma \right)\sim N\left(0,A\sigma_{A_{\log\left(\gamma \right)}}^{2}+I\sigma_{E_{\log\left(\gamma \right)}}^{2}\right)$, where **γ** is the vector of *γ*‐values, **A** the pedigree relationship matrix among animals, and **I** an identity matrix (note that, as common in animal breeding, *A* denotes a breeding value, whereas **A** denotes the relationship matrix).

**TABLE 1:** Overview of fitted models.

Model	Fixed	Random		
1	Group, time	$A_{\log_{\gamma }}$	$E_{\log_{\gamma }}$	group.time
2	Group	$A_{\log_{\gamma }}$	$E_{\log_{\gamma }}$	group.time
3		$A_{\log_{\gamma }}$	$E_{\log_{\gamma }}$	group.time
4		$A_{\log_{\gamma }}$	$E_{\log_{\gamma }}$	

*Note:* Explanation of symbols—$A_{\log_{\gamma }}$, additive genetic effect on log susceptibility; $E_{\log_{\gamma }}$, permanent environmental effect on log susceptibility.

Note that although we did not simulate any systematic group effects nor time effects, we evaluate fitting a fixed group effect in models 1 and 2 and a random interaction effect between group and time in models 1 to 3. The motivation for this is mainly found in Biemans et al. (2019), where a significant group.time variance was estimated, even with fitted fixed group and time effects (similar to our model 1). Furthermore, in empirical data, it is beforehand not known whether non‐genetic differences between group and time intervals account for a substantial part of the observed variation, thus it seems reasonable to evaluate the effect of fitting a term for time period and group in the model. Next to the genetic term for susceptibility, all models contained a permanent individual random effect, because we repeatedly observed the infection state of each individual (until it got infected).

### 2.4 Investigated Scenarios

We simulated different scenarios to investigate the performance of the GLMM in estimation of genetic parameters and breeding values for susceptibility. The varied factors were related to data design, that is, sampling interval and group size, and biological inputs, that is, the transmission rate parameter (β), and the phenotypic and genetic variance in susceptibility. For each of the variables we defined a default value, and next varied one variable at a time, while the others maintained their default value. Table 2 gives an overview of the scenarios.

**TABLE 2:** Overview of scenarios.

Variable	Default value(s)	Other simulated values
Sampling interval (Δt; days)	1	3, 5, 10, 15, 20, 30
Group size (*N*)	24 (200 groups)	12 (400), 48 (100), 96 (50), 240 (20), 480 (10)
Beta $\left(\beta ;\;\mathrm{da}y^{-1}\right)$ ^a^	0.2	0.12, 0.15, 0.4, 0.5, 1
Duration of infectious period (days)	10	—
Phenotypic variation in log susceptibility ($\sigma_{P_{\log\left(\gamma \right)}}^{2}$)	0.25	0.1, 0.5, 1
Additive genetic proportion of variance in log(γ), $a_{\log\left(\gamma \right)}^{2}$ ^b^	0.5	0, 1

^a^ For all scenarios, the recovery rate parameter, α, equalled 0.1, so that the average duration of the infectious period was 10 days. Therefore, $R_{0}\approx \beta /\alpha =10\beta$, indicating that this range for *β* corresponds to a range for $R_{0}$ from 1.2 through 10.

^b^ $a_{\log\left(\gamma \right)}^{2}=$ $\sigma_{A_{\log\left(\gamma \right)}}^{2}/\sigma_{P_{\log\left(\gamma \right)}}^{2}$. The simulated values of phenotypic variation and the proportion of genetic variance correspond to maximum values of observed‐scale heritability (Bijma et al. 2022) between 0.0125 and 0.125, the default scenario to 0.03.

For the sampling interval the approach was slightly different. There, we tested multiple values in each scenario, to see if the other variables affected the optimum sampling interval. Faster epidemics, for example, represented by higher values of β, may need shorter sampling intervals to get accurate estimates for susceptibility.

Group size was not expected to have a large effect on accuracy of estimation for susceptibility (Velthuis et al. 2007; Anacleto et al. 2015). However, by simulating different group sizes, we want to provide insights into estimation of susceptibility both from experimental (i.e., small groups) and field data (large groups). An important limitation of small groups might be the risk of stochastic fade‐out of the epidemic. This is less likely to occur if the epidemic is kick‐started by a large number of seeder individuals. However, for these seeders no information on susceptibility can be recorded, which might negatively affect accuracy of estimation (e.g., with group size 12, one‐third of the population is a seeder in our simulations).

Empirical estimates for genetic and phenotypic variance in susceptibility, as defined above, are scarce. However, for endemic infections that follow an SIS model, Hulst et al. (2021) showed that observed‐scale heritability estimates of 0.02 and 0.05 for binary infection state from a linear model, which are common values, correspond to a genetic variation in log‐susceptibility between 0.10 and 0.25. Thus, we simulated a genetic variance of 0.125 in our default scenario, and simulated a range of values around that. Together with the different simulated proportions of additive genetic variance ($a_{\log\left(\gamma \right)}^{2}$) these will give an idea of the effect of genetic and non‐genetic variation on the precision of estimates.

Given that we simulated a fixed mean duration of the infectious period of 10 days, variation in infection transmission was simulated through the transmission parameter (*β*) only. Our default value of β=0.2/day corresponds to a basic reproduction ratio ($R_{0}$) of 2. From Bijma et al. (2022) we know that genetic variation is larger at lower $R_{0}$, because indirect genetic effects are (much) larger at lower $R_{0}$. Note that a β below 0.1/day would result in an $R_{0}$ below 1, and thus in no outbreak of infection. All our simulated values are therefore above 0.1/day.

### 2.5 Comparison to a Linear Mixed Model and a ‘Threshold Model’

To compare the performance of the GLMM presented above to more classical quantitative genetic models, we fitted a classical linear mixed model (LMM) and a threshold model (TM) to the data of the default scenario (Table 2). Though a threshold model is equivalent to a GLMM with a probit link function, we use the term ‘threshold model’ because this term is well‐known in animal breeding. Therefore, in the remainder of this manuscript, the term ‘GLMM’ will refer to the GLMM with a complementary log–log link function as described above, whereas the term ‘Threshold Model’ will refer to the classical threshold model (i.e., a GLMM with a probit link).

To investigate the impact of the sampling interval on the performance of these models, we also fitted them to the scenario with a sampling interval of 10 days (all other variables as default). Both models contained a fixed interaction effect for group and time, and random genetic and permanent environmental effects. The LMM was:

$$C_{\mathrm{ikt}}=\mu +\text{Grou}p_{k}.\mathrm{Tim}e_{t}+A_{i}+E_{P,i}+e_{\mathrm{ikt}}$$

where $C_{\mathrm{ikt}}$ is a binary variable indicating whether susceptible animal *i* in group *k* got infected (1) or not (0) in interval *t* (i.e., is a case), μ is an intercept and gives the mean proportion of cases in the population, $\text{Grou}p_{k}.\mathrm{Tim}e_{t}$ is the fixed interaction between group and time, $A_{i}$ is a random genetic effect reflecting the susceptibility of individual *i*, with underlying pedigree relationship matrix, $E_{P,i}$ the random permanent effect for susceptibility of individual *i*, and $e_{\mathrm{ikt}}$ the residual term. Note that, even though $A_{i}$ and $E_{P,i}$ reflect the effect of the susceptibility of *i* on $C_{\mathrm{ikt}}$, they cannot simply be interpreted as estimates of susceptibility in the strict epidemiological sense (i.e., *γ*), because there is no direct relationship of the LMM to the transmission model (Equation 1). For this reason, $A_{i}$ and $E_{P,i}$ do not have subscript *γ* or $\log_{\gamma }$.

The threshold model contained the same explanatory variables as the LMM:

$$\text{Probit}\left(P_{\mathrm{ikt}}\right)=\mu +\text{Grou}p_{k}.\mathrm{Tim}e_{t}+A_{i}+E_{P,i}$$

Here, the response variable $\text{Probit}\left(P_{\mathrm{ikt}}\right)$ indicates the probit‐transformed probability of infection of susceptible individual *i*. Note that the threshold model is in fact a GLMM with a probit link function and a binomial distribution. However, as this model is historically known as the threshold model in animal breeding, we will use that name here, and reserve the term GLMM for the model based on the transmission process (model 1 and Table 1). The fixed Group_Time effect in the LMM and the TM has a similar role to the offset in the GLMM, but it has an unknown value and is thus estimated from the data, whereas the offset in the GLMM is based on the known exposure.

The GLMM, LMM and TM were used to estimate genetic variance and breeding values, which were then compared to the simulated true values. Note that the LMM and TM will give estimates on a scale different from the $\log\left(\gamma \right)$ scale used in the simulation and the GLMM. So some ‘bias’ in estimated genetic parameters and dispersion of EBV is expected. Nevertheless, we expect the EBV from the LMM and TM to capture genetic variation in susceptibility, which we will measure by their correlation with the true breeding values for $\ln\left(\gamma \right)$.

## 3 Results

We start this section by comparing the precision and bias of parameter estimates for the four GLMMs, under the default scenario and for a range of sampling intervals (Tables 1 and 2). Based on this comparison, we choose the best performing GLMM to investigate the effect of the different variables on parameter estimation and accuracy of breeding values, and then compare these to the output of the LMM and threshold model. All estimates are the mean of 100 replicates, with corresponding standard errors. Full results are available in Supporting Information.

### 3.1 Comparison of Models 1 Through 4

For the default scenario, there was a considerable difference in accuracy of estimation of β between models 1 through 4 (Table 3). Model 1 considerably underestimated β, whereas models 3 and 4 yielded unbiased and accurate estimates of β. This indicates that models 3 and 4 are well able to capture the mean level of transmission, while this is less the case for models 1 and 2. Although the estimate from model 2 was not significantly biased, the standard error on β for model 2 was relatively large compared to those for model 3 and 4.

**TABLE 3:** Mean estimates ± SE of epidemiological parameters and variance components obtained by models 1 through 4 for the default scenario.

Model	β (0.20)	$\sigma_{A_{\gamma }}^{2}$ (0.125)	$\sigma_{E_{\gamma }}^{2}$ (0.125)	$\sigma_{\mathrm{GT}}^{2}\;\left(0\right)$
1	0.14 ± 0.008	0.201 ± 0.0060	1.117 ± 0.0110	0.10 ± 0.004
2	0.19 ± 0.012	0.143 ± 0.0043	0.359 ± 0.0074	0.14 ± 0.004
3	0.20 ± 0.001	0.124 ± 0.0034	0.150 ± 0.0052	0.14 ± 0.004
4	0.20 ± 0.001	0.125 ± 0.0034	0.177 ± 0.0053	NA

*Note:* Explanation of symbols—β, transmission rate parameter; $\sigma_{A_{\gamma }}^{2}$, additive genetic variance in log‐susceptibility; $\sigma_{E_{\gamma }}^{2}$, permanent environmental variance in log‐susceptibility; $\sigma_{A_{\mathrm{GT}}}^{2}$, group × time variance in log‐susceptibility. True values are in brackets.

For the genetic variance of susceptibility, the results were similar (Table 3). Model 1 considerably overestimated the genetic variance, and models 1 and 2 had slightly larger standard errors than models 3 and 4. Also for the permanent environmental variance the pattern was similar, but more pronounced: overestimation in model 1, less so in model 2, while models 3 and 4 resulted in best and most accurate estimates. These results show that including fixed effects for group and particularly for time apparently leads to biased and inaccurate estimates of variances and β, even though the fixed effects were not statistically significant in any of those models.

Even though a random group.time interaction was not simulated, all models that included this term gave significant estimates for its variance. This suggests that such variance arises in the stochastic transmission processes, and accounts for a significant part of the variation in *y*. Omitting the group.time random effect resulted in a slightly increased estimate for the permanent environment effect (models 3 vs. 4).

**FIGURE 2:**
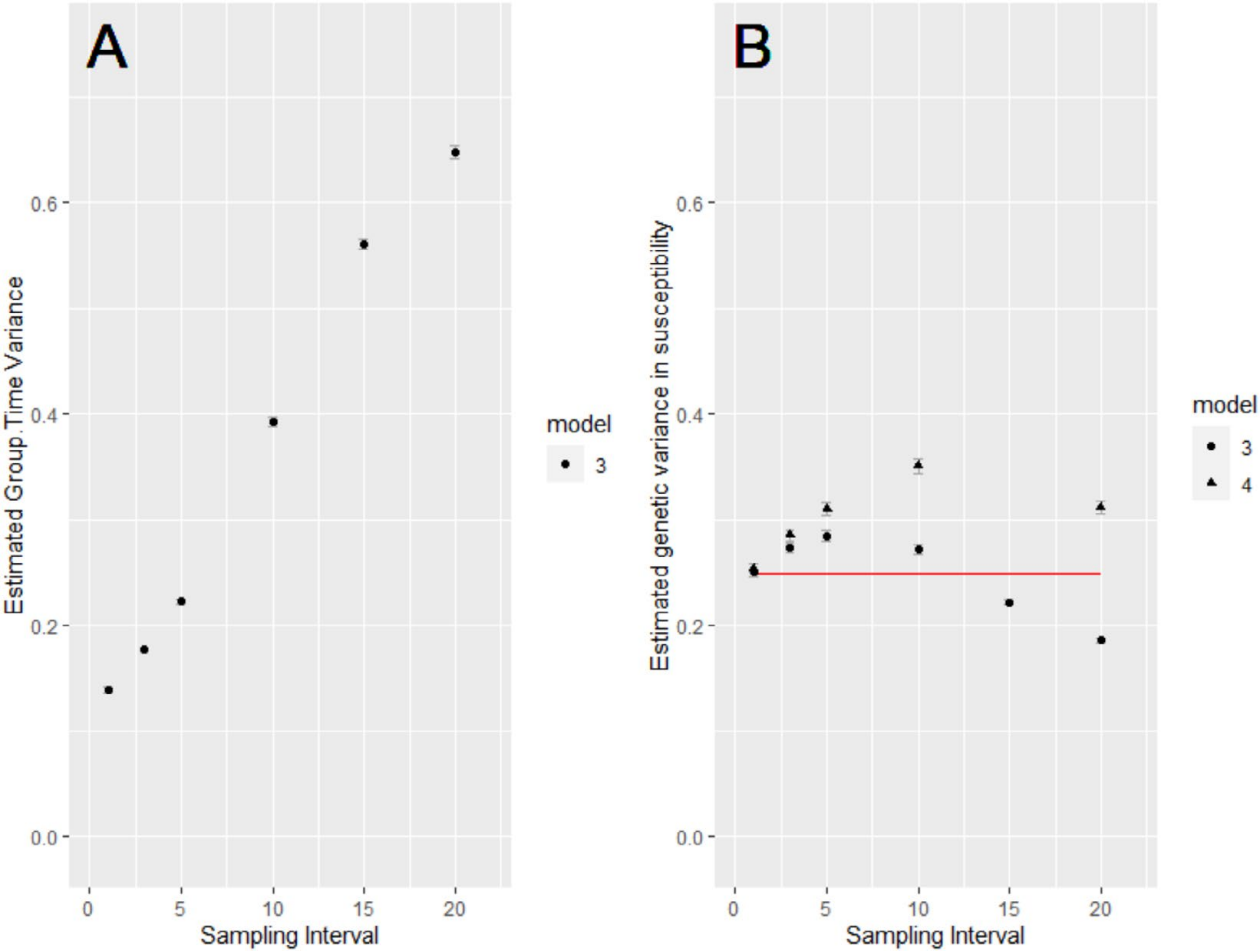
(A) Estimated group.time variance for different sampling intervals. (B) Estimated genetic variance in susceptibility for different sampling intervals, estimates from models 3 and 4. The red line indicates the simulated (i.e., true) genetic variance, and error bars indicate the SE. All other input values as in the default scenario. [Colour figure can be viewed at wileyonlinelibrary.com]

The estimated group.time variance increased considerably with longer sampling intervals (Figure 2A). A comparison of model 3 and model 4 (Figure 2B) shows that, if a group.time interaction is not included (model 4), part of its variance ends up in the estimate for the genetic variance, leading to overestimation of this variance with larger sampling intervals. Addition of a group.time random term to the GLMM is thus especially important when the sampling interval is relatively large. Since the other estimates were not significantly affected by the group.time variance with smaller intervals (see results for models 3 and 4 for an interval of 1 day in Figure 2B), it seems advisable to add a group.time effect to the model for any sampling interval.

Based on these results, we decided to fit a GLMM without fixed group and time effects and with a random group.time effect (model 3), and we will show estimates from this model in the remainder of the Results.

### 3.2 Sampling Interval

**FIGURE 3:**
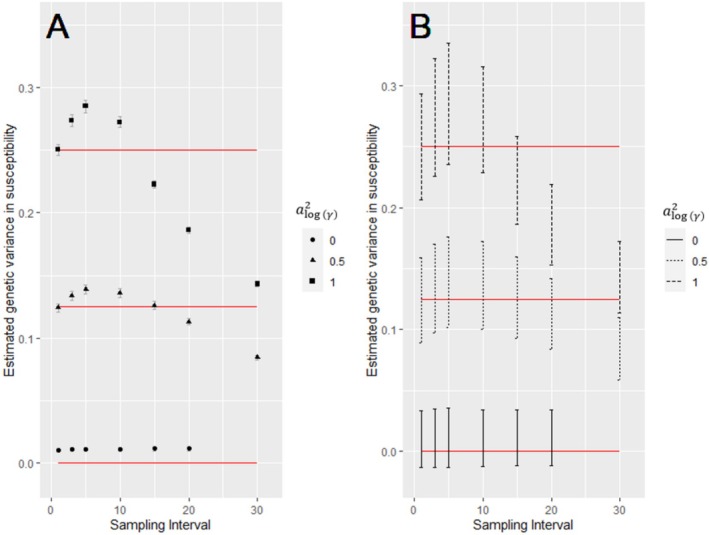
Mean estimates with SE of genetic variance in susceptibility (panel A) and mean 95% confidence intervals of these estimates (panel B) for different sampling intervals and three values of the proportion of additive genetic variance ($a_{\log\left(\gamma \right)}^{2}$), from model 3. Other variables as in the default scenario. Red lines indicate the simulated genetic variance, grey error bars in panel A indicate standard errors of the mean of 100 replicates. Note the missing estimate for interval 30, $a_{\log\left(\gamma \right)}^{2}$ = 0, due to convergence problems. [Colour figure can be viewed at wileyonlinelibrary.com]

Figure 3A shows that the genetic variance in susceptibility was slightly overestimated if there was no genetic variance simulated ($a_{\log_{\gamma }}^{2}=0$). However, this was expected since variance estimates were forced to be non‐negative in ASREML. In the absence of genetic variation, the sampling interval did not affect the estimates of the genetic variance. When genetic variance was simulated, only a sampling interval of 1 day resulted in an unbiased estimate. For sampling intervals of 3, 5, and 10 days the variances were slightly overestimated, while for sampling interval above 15 days, they were underestimated. The largest overestimation occurred for an interval of 5 days, and the largest underestimation for an interval of 30 days. Especially for a simulated genetic variance of 0.25 ($a_{\log_{\gamma }}^{2}=1$), the underestimation with sampling intervals of 20 and 30 days was considerable. The precision of estimates was quite similar across sampling intervals, with 95% confidence intervals covering a range of 0.08 for $a_{\log_{\gamma }}^{2}=1$, 0.07 for $a_{\log_{\gamma }}^{2}=0.5$, and 0.05 for $a_{\log_{\gamma }}^{2}=0$ (Figure 3B).

### 3.3 Simulated Phenotypic Variance

**FIGURE 4:**
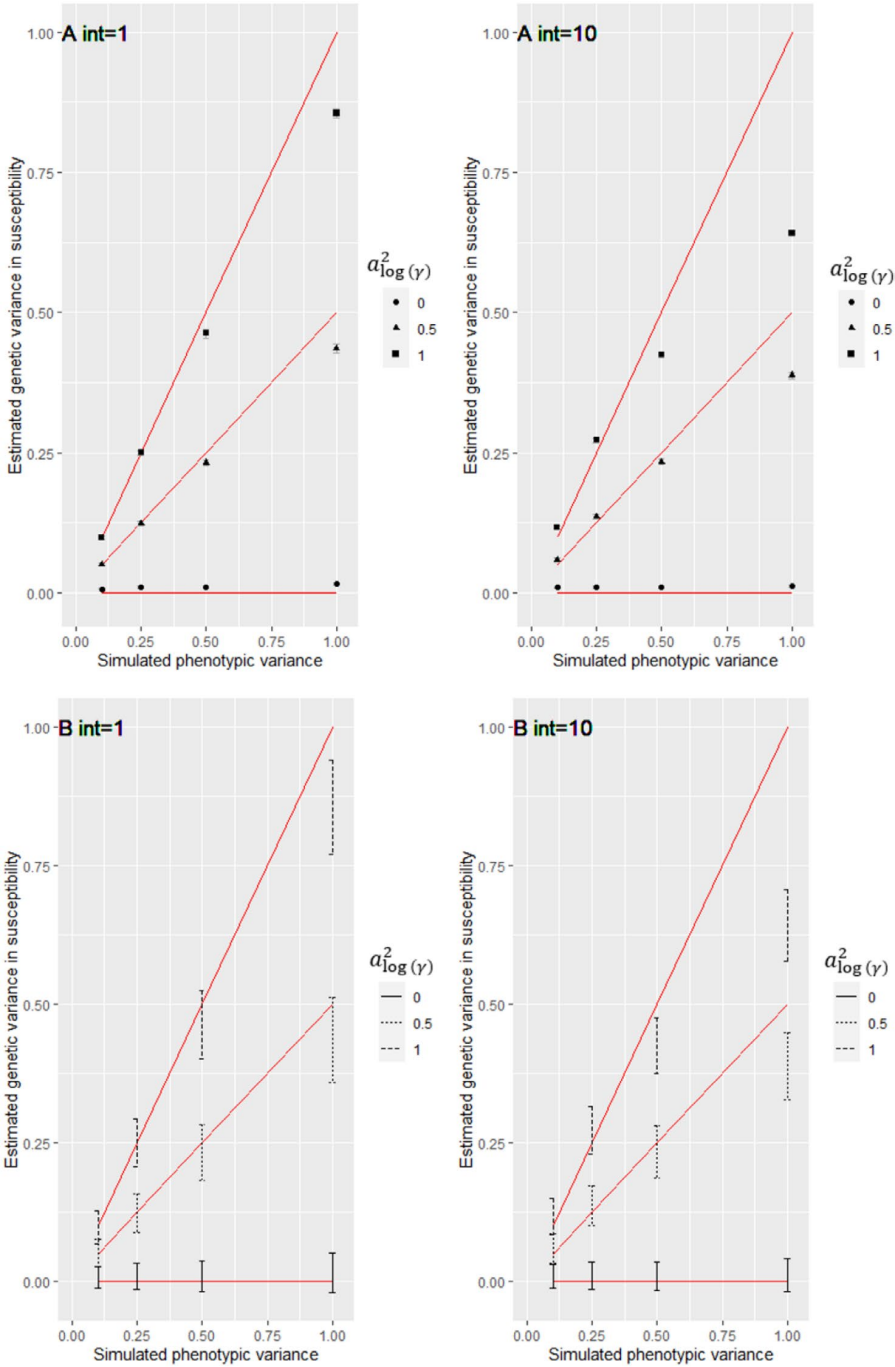
Mean estimates with SE of genetic variance in susceptibility (panel A) and mean confidence intervals of these estimates (panel B) for different simulated values of phenotypic variance in log‐susceptibility, three values of the proportion of additive genetic variance, and sampling interval of 1 day (left panel) and 10 days (right panel). Other variables as in the default scenario. Red lines indicate the simulated genetic variance, grey error bars in panel A indicate standard errors of the mean of 100 replicates. [Colour figure can be viewed at wileyonlinelibrary.com]

Figure 4A shows that the GLMM picks up the trend in simulated phenotypic variance and the proportion of additive genetic variance in susceptibility quite well for a sampling interval of 1 day. With this sampling interval, estimates of the genetic variance were unbiased for true values of 0.25 and lower. For a sampling interval of 10 days, the genetic variance was underestimated when phenotypic variance and/or heritability were large. Again, the presence of genetic variation was accurately identified (i.e., confidence interval overlapped zero only when the true variance was zero). Absolute precision decreased with increased simulated genetic variance (Figure 4B). However, relative to the size of the estimate, precision increased.

### 3.4 Transmission Rate Parameter (β)

**FIGURE 5:**
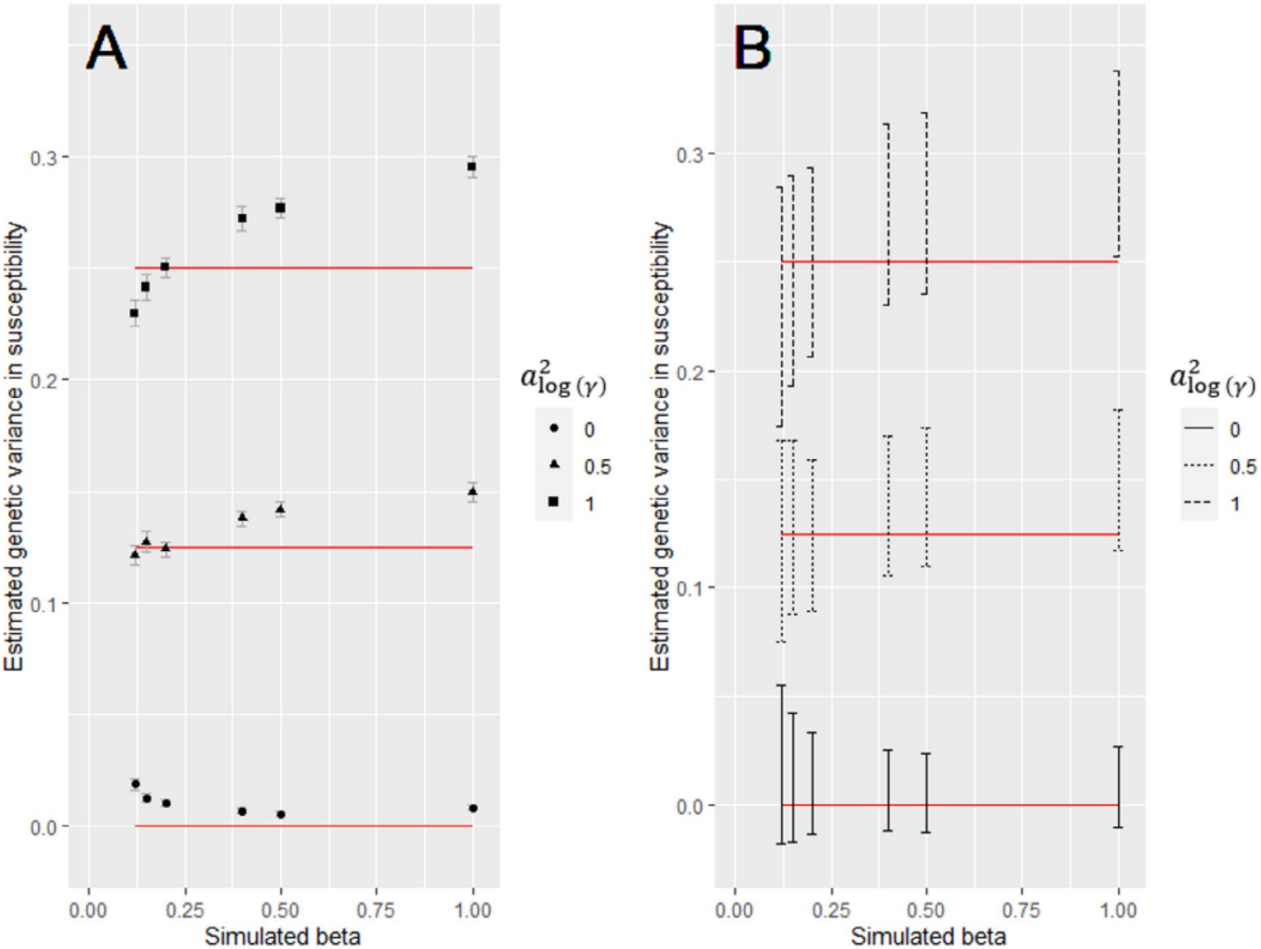
Mean estimates with SE of genetic variance in susceptibility (panel A) and mean confidence intervals of these estimates (panel B) for different simulated values of the average transmission rate parameter β and three values of the proportion of additive genetic variance ($a_{\log\left(\gamma \right)}^{2}$). Other variables as in the default scenario. Red lines indicate the simulated genetic variance, grey error bars in panel A indicate standard errors of the mean of 100 replicates. [Colour figure can be viewed at wileyonlinelibrary.com]

Figure 5A shows the effect of the transmission rate parameter, β, on genetic variance estimates. Higher values of β (and thus higher $R_{0}$ since we used a constant recovery rate parameter), result in epidemics where transmission goes faster, so that more individuals get infected per unit of time, and a greater proportion of individuals who become ultimately infected. Higher values of β generally led to increased overestimation of the genetic variance in susceptibility. However, the pattern was different in the absence of genetic variance (simulated heritability of zero), where overestimation decreased with increasing β. With $a_{\log_{\gamma }}^{2}=1$ and a genetic variance 0.25, low values of β resulted in a slightly underestimated genetic variance. Nevertheless, deviations from the simulated value were relatively small, except for β=1 ($R_{0}=10$, reflecting an outbreak where nearly all individuals become infected). Precision of estimates increased slightly with β, and was lower for a β of 0.12 and 0.15 (Figure 5B).

### 3.5 Group Size

**FIGURE 6:**
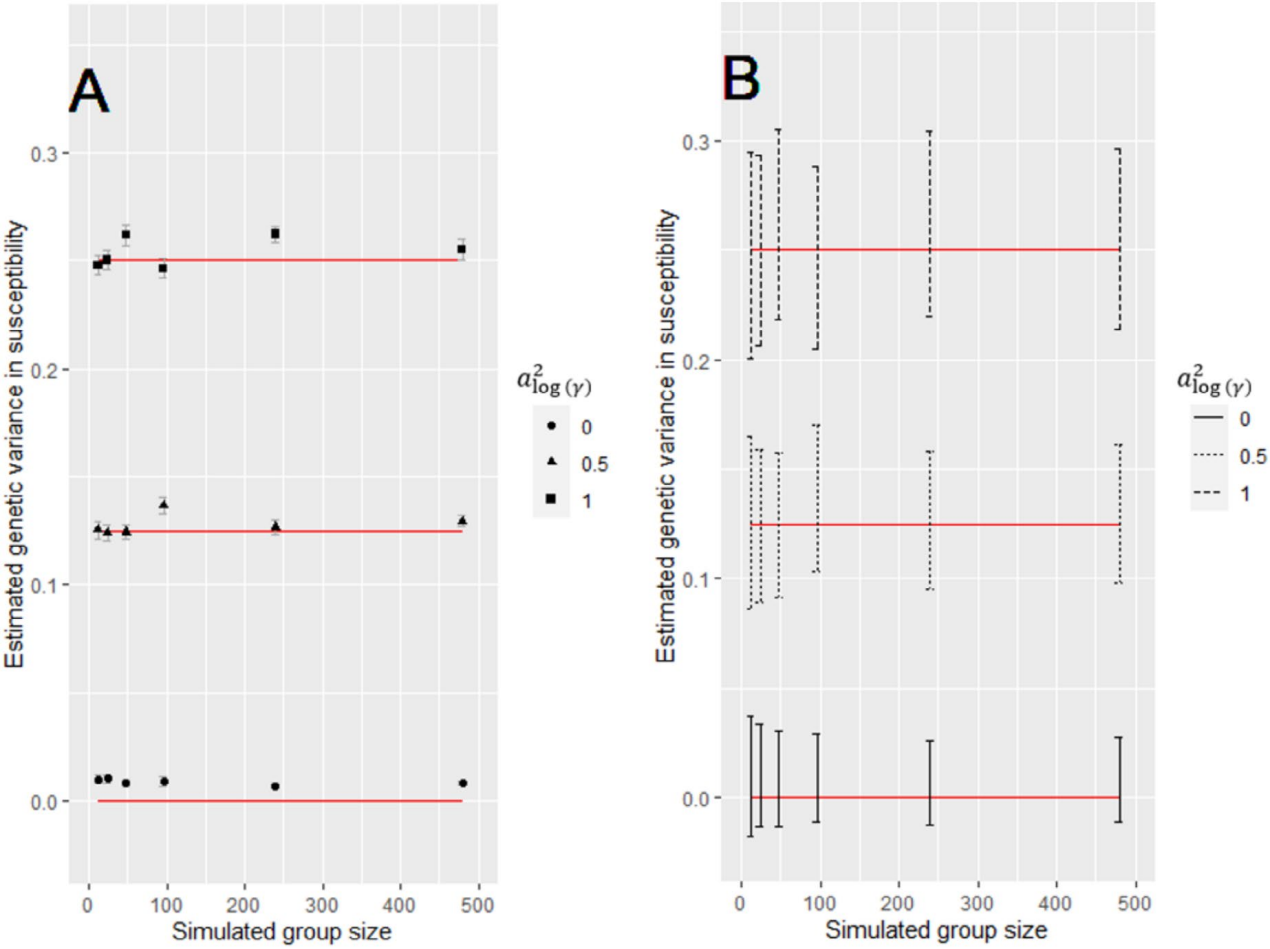
Mean estimates with SE of genetic variance in susceptibility (panel A) and mean confidence intervals of these estimates (panel B) for different simulated group size and three values of the proportion of additive genetic variance ($a_{\log\left(\gamma \right)}^{2}$). Other variables as in the default scenario. Red lines indicate the simulated genetic variance, grey error bars in panel A indicate standard errors of the mean of 100 replicates. [Colour figure can be viewed at wileyonlinelibrary.com]

Figure 6 shows that, at the same total number of individuals, group size had a minor effect on the bias and precision of estimates of genetic variance in susceptibility. There seemed to be a little overestimation for some group sizes, but no clear pattern was observed. The precision of the estimates was slightly lower for group size 12 (Figure 6B), which is likely caused by the relative large proportion of seeder individuals (without observation) in that scenario.

### 3.6 Accuracy and Dispersion of Estimated Breeding Values

**FIGURE 7:**
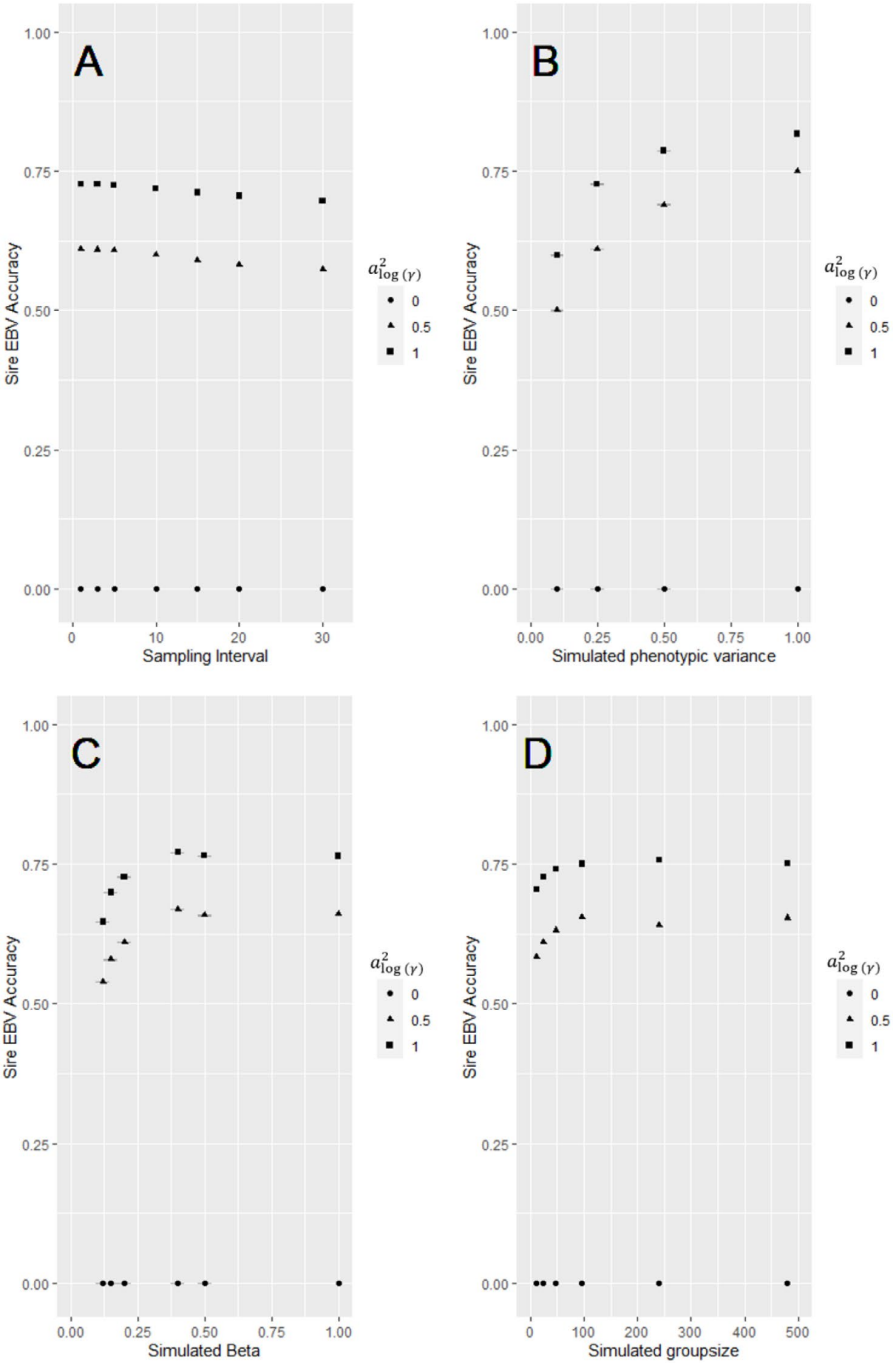
Accuracy of sire estimated breeding values (EBV), for three values of the proportion of additive genetic variance ($a_{\log\left(\gamma \right)}^{2}$). (A) For different sampling intervals. (B) For different phenotypic variances in log‐susceptibility. (C) For different betas. (D) For different contact group sizes. Other values as in the default scenario (Table 2). [Colour figure can be viewed at wileyonlinelibrary.com]

Figure 7A shows the accuracy of estimated breeding values (EBVs) for the logarithm of susceptibility of sires, being the correlation between true and estimated breeding sire values. Despite the low observed‐scale heritability of binary disease state, which was either 0.02 or 0.05 (Table 2), the accuracies of EBVs for susceptibility suggest that meaningful response to selection can be obtained for the scenarios investigated here. Accuracy was little affected by the sampling interval. As expected, a higher proportion of additive genetic variance ($a_{\log_{\gamma }}^{2}$) led to higher accuracy. For a constant $a_{\log_{\gamma }}^{2}$, accuracy increased with the phenotypic variance in log‐susceptibility, and thus with the genetic variance in log‐susceptibility (since $\sigma_{A_{\log_{\gamma }}}^{2}=a_{\log_{\gamma }}^{2}\sigma_{P_{\log_{\gamma }}}^{2}$; Figure 7B). At first glance, this result may seem surprising, since EBV accuracy for ordinary quantitative traits depends only on heritability, not on the level of the genetic variance. However, greater additive genetic variance for log‐susceptibility creates greater observed‐scale heritability (equations 10c and 36 in Bijma et al. 2022), resulting in higher accuracy of EBVs.

Accuracy increased with the transmission rate parameter, but reached a plateau for higher values of β (Figure 7C). An intuitive explanation is that outbreak size is smaller with lower β (Figure 1), leaving a larger fraction of the population uninfected. There is no difference in information for susceptibility between these uninfected individuals, thus breeding value estimation will be less accurate if a larger fraction of the population stays uninfected. The effect of group size on accuracy was similar as that of β (Figure 7D). In our design, each contact group had four seeder individuals that started the infection, irrespective of group size. Thus, the remaining number of individuals for which meaningful records for susceptibility could be obtained, was larger in the larger contact groups, just because the proportion of seeder individuals was lower in those groups.

Next to the accuracy of sire EBVs, we also calculated accuracy for dam and offspring EBVs (see Supporting Information: Results). The patterns for dam and offspring EBV accuracy were very similar to those in Figure 7, and align fully with our expectations based on population structure. Our population was simulated according to a full‐sib half‐sib design, with 50 offspring per sire, 10 per dam, and records only on the offspring. Therefore, the accuracy of offspring EBVs was higher than that of dam EBVs, because each offspring had, next to its own records, information on 4 full‐sibs and 45 half‐sibs, whereas dams only had information from 10 offspring.

**FIGURE 8:**
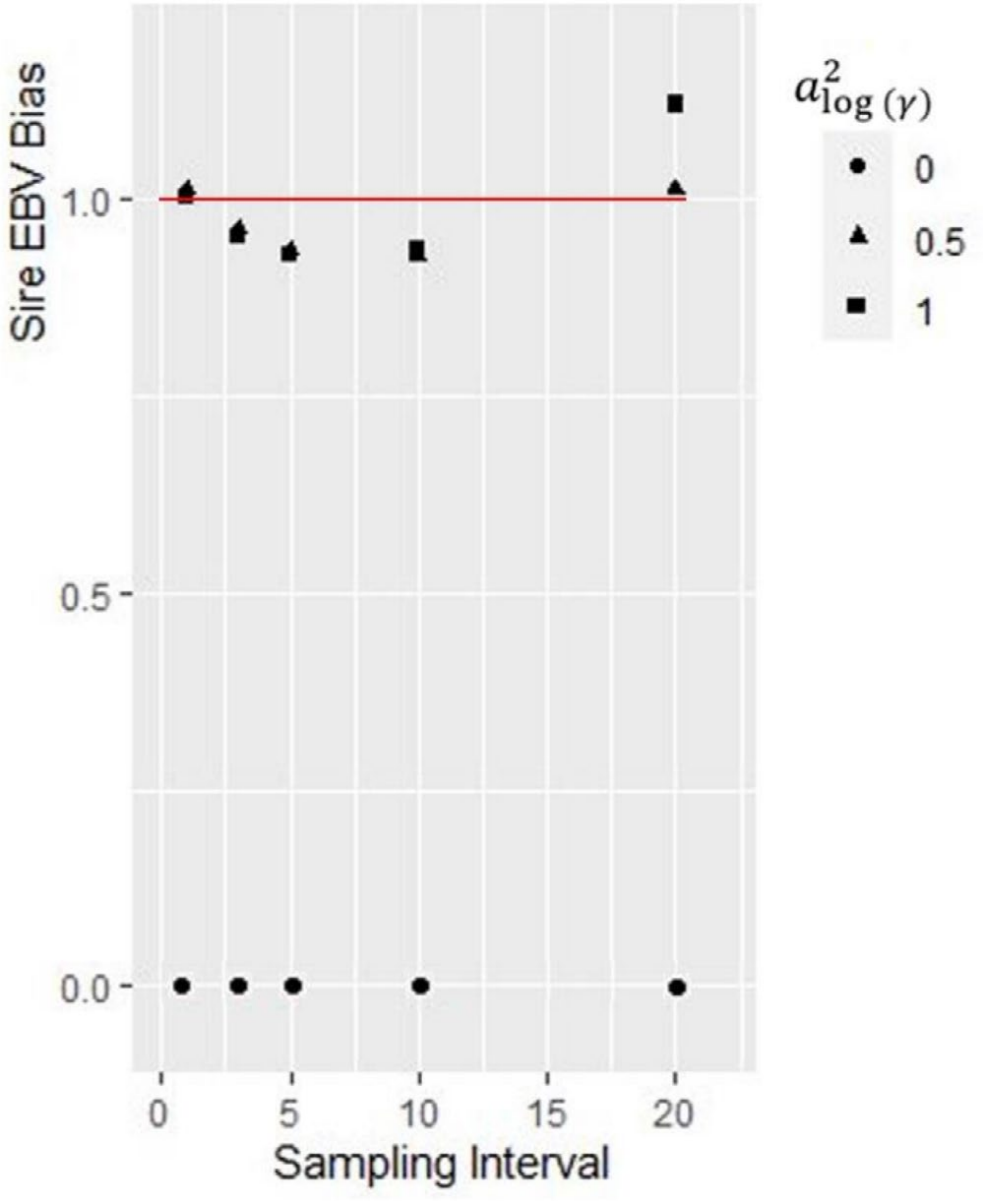
Dispersion of EBVs as measured by the regression coefficient of true sire breeding values on estimated sire breeding values for the logarithm of susceptibility. For different sampling intervals. Other values as in the default scenario. A value of 1 means correct dispersion of EBVs, a value > 1 means underdispersion of EBVs, and a value < 1 means overdispersion of EBVs. [Colour figure can be viewed at wileyonlinelibrary.com]

Figure 8 shows the dispersion of sire EBVs for the logarithm of susceptibility, as measured by the regression coefficient of true breeding values on EBVs (sometimes referred to as ‘bias’ of EBV). A regression coefficient equal to one indicates a correct dispersion of EBVs. Values smaller than one indicate that EBVs require shrinkage to match the dispersion of the true BVs, and thus imply overdispersion of EBVs. Analogously, values greater than one indicate that EBVs require upscaling, and thus imply underdispersion of EBVs. The pattern in dispersion of EBVs reflects that of the genetic variance estimates (Figure 3), with correct dispersion for an interval of 1 day, overdispersion of EBVs for intervals of 3 to 10 days, and underdispersion of EBVs for intervals of 15 days and more. This pattern was found for all scenarios investigated above.

### 3.7 Comparison to LMM and Threshold Model

**FIGURE 9:**
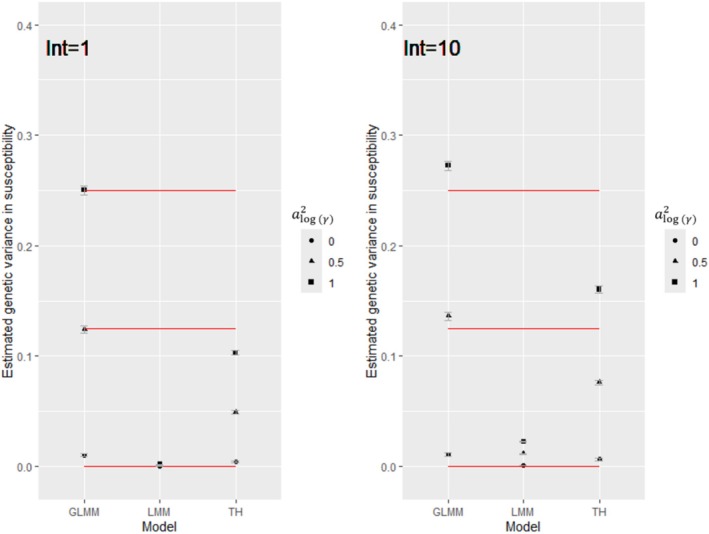
Estimated genetic variances from the GLMM, LMM, and TM, for a sampling interval of 1 day (left panel) and 10 days (right panel). Note that, for the LMM and an interval of 1 day (left panel), all three estimates were close to zero and therefore difficult to distinguish in the figure. Estimates for the TM are on the underlying liability scale. [Colour figure can be viewed at wileyonlinelibrary.com]

Figure 9 shows genetic variance estimates for the LMM and the TM (values for the TM are presented on the underlying liability scale, not transformed to the observed scale). These two models yielded a genetic variance estimate that is considerably smaller than the genetic variance in log‐susceptibility. These results illustrate that the scale of the LMM and the TM is totally different from that of log‐susceptibility, and the epidemiological interpretation of these estimates is not immediately clear. As with the GLMM, the estimates increased with longer sampling intervals. However, the relative increase was larger for the LMM and TM.

**FIGURE 10:**
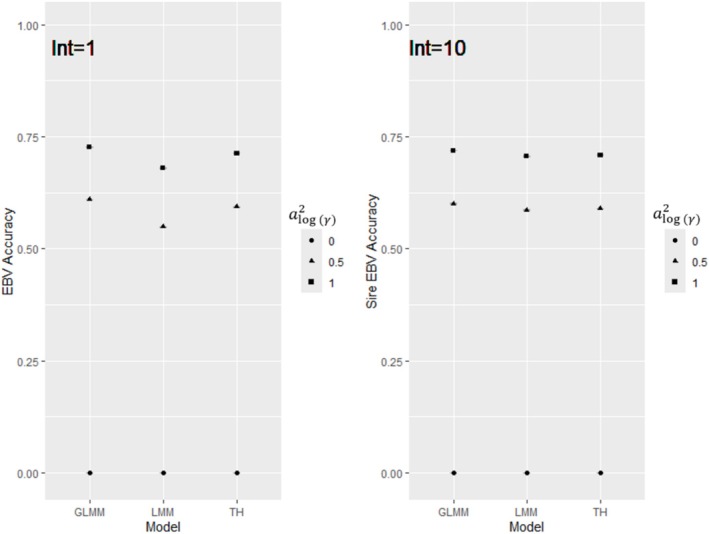
Accuracy of Sire EBVs for susceptibility obtained from GLMM, LMM, and TM for sampling interval of 1 day (left panel) and 10 days (right panel). [Colour figure can be viewed at wileyonlinelibrary.com]

Figure 10 shows the accuracy of Sire EBVs for the three models. The accuracy is a little higher for the GLMM compared to the LMM and the T, and the difference in accuracy is smallest for a sampling interval of 10 days. Hence, despite the very different scales of the estimates (Figure 9), differences in accuracy are relatively small.

## 4 Discussion

Here we showed that a GLMM founded in epidemiological theory is able to produce accurate and almost unbiased estimates of the genetic variance for susceptibility to an infection, when this infection follows an SIR model with genetic variation in susceptibility only, and when dense longitudinal records of individual infection status are available. The sampling interval was the main factor affecting the quality of the estimates, especially with a larger simulated genetic variance. In general, the shorter the sampling interval, the better the estimates.

The sampling interval is important, because it determines the quality of the information. The *y*‐variable for the GLMM were binary records on whether (1) or not (0) individuals, who were susceptible at the start of an observation interval, became infected during that interval. Hence, in contrast to the common analysis in animal breeding, our *y*‐variable was not the binary final infection status of an individual, but indicated whether or not an initially non‐infected individual became infected in the observation interval in question. To accurately determine the susceptibility of an individual, each observation was corrected for the exposure to infectious individuals by including an offset in the model, which was based on the fraction of infected group mates at the start of the interval (the $I\left(t\right)/N$ in Equation 4). However, if the sampling interval becomes too large, the information in the offset gets outdated, either because additional individuals became infected after the start of the interval, resulting in an offset that underestimates exposure, or because individuals recovered before the end of the interval, giving an offset that overestimates exposure. Furthermore, a larger sampling interval will result in less accurate information on the (differences in) infection times of individuals. For example, in a very long interval, nearly all individuals may become infected, making it impossible to discriminate between them. The group.time random effect captures (part of) the extra variation that arises due to the outdated offset (see Figure 2), because this effect is estimated from the actual observations in the interval (i.e., the number of new infections) and not, like the offset, based on the fraction of infected individuals at the start of the interval.

Coincidentally, we found that a sampling interval of 1 day yielded good estimates. This result, however, is not general, but will depend on the rate at which transmission and recovery events proceed for the specific infection. To get more grip on the sampling interval needed for accurate estimation of genetic parameters, we calculated the minimum expected interval between events, events being infection of a susceptible individual and recovery of an infected individual. The minimum expected interval depends on the total rate (TR) with which events happen in the group. The total rate is the sum of the transmission rate in the group, Nβsi, and the recovery rate in the group, Nαi,

$$\mathrm{TR}=N\left(\beta \;s\;i+\alpha \;i\right)$$

where *N* is the group size, β the transmission rate parameter, α the (average) recovery rate parameter, *s* the fraction of individuals in the group that is susceptible, *s* = *S*/*N*, and *i* the fraction of individuals in the group that is infected, *i* = *I*/*N* (as usual, *S* and *I* denote the absolute numbers of susceptible and infected individuals in the group; the expression for the TR here ignores genetic differences among individuals).

As is clear from the *N* in the expression for the TR, the absolute TR is proportionally larger in larger groups. The accuracy of estimates, however, likely depends on relative errors, that is, errors in the fractions *s* and *i*, rather than on the absolute values *S* and *I*. This is also suggested by the very minor effect of group size on the accuracy and dispersion of estimates (Figure 6). For this reason, we focus on the total rate per individual, TR/N=βsi+αi.

Furthermore, the event rate in an outbreak changes over time; it is initially slow when only few individuals are infected, then increases to a maximum, and finally decreases until the outbreak dies out. The shortest (expected) event interval equals the reciprocal of the maximum rate,

$$\min.\;\text{expected interval}=\frac{1}{\max\left(\mathrm{TR}/N\right)}$$

**FIGURE 11:**
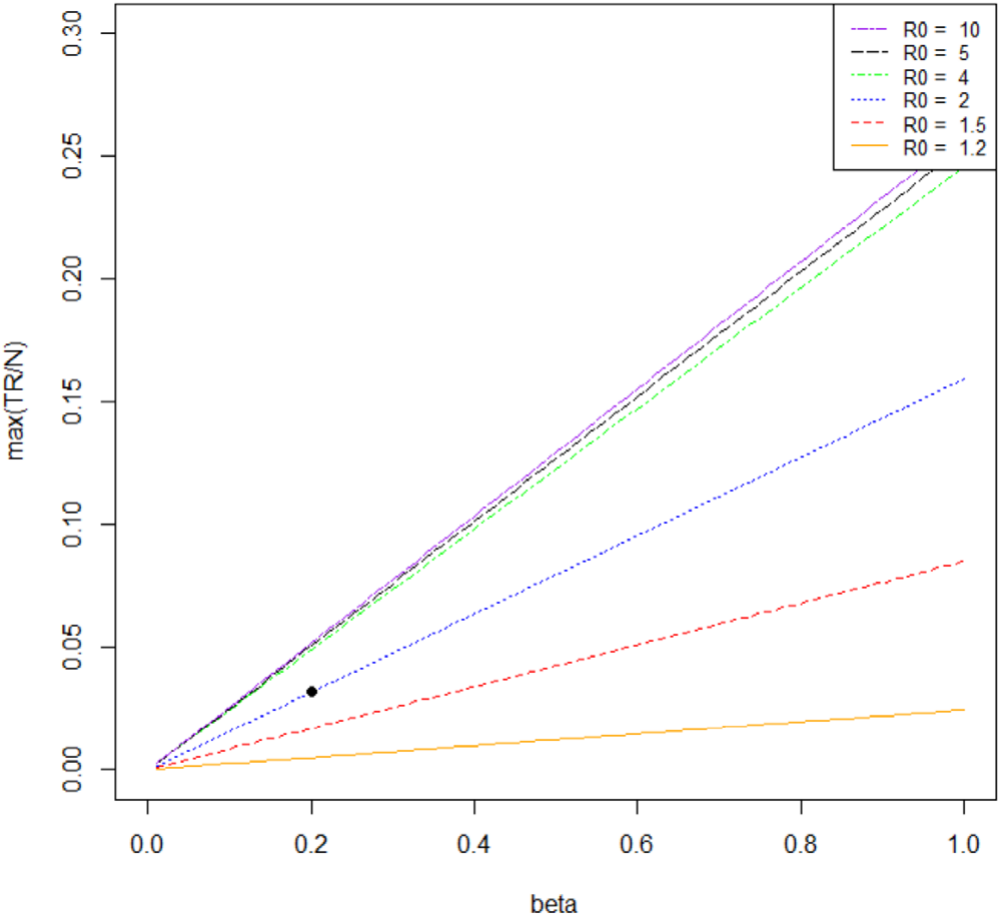
Expected maximum total rate per individual, as a function of the transmission rate parameter (*β*) and the basic reproduction number ($R_{0}=\beta /\alpha$). Note that these results do not depend on group size. The dot indicates the default scenario. [Colour figure can be viewed at wileyonlinelibrary.com]

We approximated the total rate and the corresponding minimum expected interval using the deterministic version of the SIR‐model (see Appendix A for methods). Figure 11 shows the results as a function of *β* and for different values of $R_{0}$. These two parameters also capture variation in the recovery rate parameter, which can be found as $\alpha =\beta /R_{0}$.

Our default scenario had a sampling interval of 1 day and resulted in an almost unbiased estimate of the genetic variance in susceptibility. This scenario has *β* = 0.2/day, $R_{0}$ = 2, and *N* = 24 individuals, corresponding to a maximum total rate per individual of 0.032/day, a maximum total rate in the entire group of 0.77/day, and a (expected) minimum interval between consecutive events in the group of 1/0.77 = 1.31 day. This result suggests that, for a maximum total rate per individual of 0.032/day, a sampling interval of ~1 day suffices. For the case of *β* = 0.1/day and $R_{0}$ = 2, for example, $\max\left(\mathrm{TR}/N\right)$ = 0.016/day, suggesting a required sampling interval of approximately 0.032/0.016 × 1 day = 2 days. In other words, at the same $R_{0}$, so the same expected size of the epidemic, but half the transmission rate parameter, the epidemic proceeds at half the rate, and the sampling interval can be doubled. Analogously, for the case of *β* = 0.2/day and $R_{0}$ = 1.5, for example, we find $\max\left(\mathrm{TR}/N\right)$ = 0.017/day, suggesting a required sampling interval of approximately 0.032/0.017 × 1 day = 1.88 ≈ 2 days. These results are approximate and indicative, and a fully detailed investigation of the required sampling interval using extensive stochastic simulations will require further research. Nevertheless, the current results on the effect of the sampling interval (Figures 2 and 3) together with Figure 11 give an indication of the approximate sampling interval needed to avoid large bias in the estimated genetic variance in susceptibility estimated with a GLMM.

The model comparison (Table 3) showed that the inclusion of fixed effects for group and time in the model led to biased and inaccurate estimates for β and the genetic variance in susceptibility. The underestimation and large uncertainty in the estimate of β for these models, that is, the much larger standard errors than for the models without fixed effects, show that the models with fixed effects poorly picked up the mean level of transmission, which likely causes errors in estimation of the individual susceptibility effect and thereby probably has led to the overestimated susceptibility variance.

Offsets are commonly used in GLMM to account for known exposure, whereas linear mixed models in quantitative genetics typically include fixed effects for, for example, herd, year, and season. The essential difference is that the value of an offset is known, it is essentially a model term with a known value, whereas a fixed effect is fitted to the data. Our results indicate that using knowledge of exposure by including an offset is beneficial compared to fitting a fixed effect. The true presence of fixed effects, for example, due to herd, year, or season, on the exposure of individuals to an infection will be captured by the offset, as it will surface in the *I* of the $\log\left(I∆t/N\right)$ term in Equation (4). However, systematic effects on susceptibility, that is, on β, will only partly be captured by the offset. For example, a larger β will not only lead to increased exposure (captured by the offset), but also to a transmission rate within the interval that is greater than expected given the offset. If systematic environmental differences in β are confounded with genetic differences, such as certain families being present in certain herds or groups, this might inflate genetic variance estimates. The inclusion of a group effect (model 2) had relatively minor detrimental effects on the quality of estimates (models 2 vs. 3 in Table 3) and might be considered in such cases.

In animal breeding, the usual approach for genetic analysis of infectious disease data is to analyse binary disease/infection state or an indicator trait (e.g., somatic cell count) with LMM or GLMM‐like threshold models (e.g., Pérez‐Cabal et al. 2009; Welderufael et al. 2017; Martin et al. 2018). Importantly, these linear and threshold models are not based on epidemiological principles, and resulting estimates of genetic parameters and breeding values have no clear epidemiological interpretation. Our comparison of the estimates from a linear model (LMM), threshold model (TM) and GLMM show that the genetic variance estimates of the LMM and TM are on a totally different scale as the simulated values in susceptibility and the estimates from the GLMM. Despite this scale difference, the LMM and TM yielded only a little lower accuracy than the GLMM, which means that the ranking of the animals is similar for the three models. The scale of the EBVs from the LMM and TM, however, is very different from that of susceptibility. Hence, like the variance estimates, breeding values estimated with a LMM or TM do not predict response to selection for the infection. The estimates of the GLMM, in contrast, can be used to predict the change in the prevalence of the infection over generations and in the basic reproduction number of the infection (see also Bijma et al. 2022). The connection of the GLMM‐EBVs to response to selection also facilitates the calculation of the index‐weights in a multitrait breeding goal.

As far as we know, Biemans et al. (2019) is the only study so far that used a GLMM with an offset correcting for exposure to estimate genetic parameters for infectious disease susceptibility from real data. Corresponding to our results for the group.time effect, they found a significant farm*period variance. In contrast to our results, however, dropping this effect from the model led to a (non‐significant) decrease in estimated genetic variance in susceptibility, instead of an increase. Their estimated genetic variance of 0.55 at a sampling interval of half the infectious period (which corresponds to 5 days in our case) might be underestimated if we look at our results (see Figure 3). However, theoretical results suggest that a genetic variance in susceptibility of 0.25 is already quite large and might be at the higher end of a realistic range (Hulst et al. 2021; Bijma et al. 2022). At present, however, we still have limited information on the magnitude little of the genetic variance in susceptibility, and more empirical estimates are needed to get a better impression of realistic values.

Because diagnosis often occurs discretely over time—that is, at specific points in time rather than continuously—the precise time of infection is typically unknown. Therefore, data on individual infection status are typically binary, indicating whether an individual is infected or not, rather than a measure of time to infection. For this reason, we used a GLMM for binary data. When the time to infection is known, survival analysis might be equivalent to our analysis (e.g., Ducrocq 1994). In such an analysis, offsets could be included as time‐dependent effects with a known value, if available software (e.g., Ducrocq et al. 2010) allows this.

Anacleto et al. (2015) did a study similar to ours, with simulated infection data, but a different Bayesian method to estimate genetic parameters. They showed that group size has very little effect on prediction accuracy for susceptibility, which agrees with our results in Figure 6. Furthermore, our results align with their indication that prediction accuracy starts to decline with increasing sampling interval, in particular when the interval becomes larger than the average duration of the infectious period.

We used ASREML (Gilmour et al. 2015) for the statistical genetic analysis of the simulated binary data, a standard software widely used in animal breeding. ASREML uses the Penalized Quasi Likelihood algorithm (Schall 1991; Breslow and Clayton 1993), which avoids the problem of high‐dimensional numerical integration in the evaluation of the likelihood. Unfortunately, variance estimates from that procedure can be seriously biased, as first observed by Gilmour et al. (1985) and studied in detail by Breslow and Lin (1995), Engel (1998), and Engel and Buist (1998) among others. Engel and Buist (1998) show that the bias has a complex pattern. It is larger with less information per random effect, and thus increases with fewer observations per random effect but decreases when random effects are correlated due to genomic or family relationships. Moreover, the bias depends on the number of fixed effects in the model; it is mostly negative, but can also take positive values when the number of fixed effects is large. Due to this complex nature, there is no simple correction for the bias, particularly not for animal breeding applications where the number of fixed effects is often large (Engel and Buist 1998).

In our results, the bias was mostly positive, except when the sampling interval was long, in which case bias turned negative (Figures 2 and 3). Moreover, the number of fixed effects fitted for group had almost no effect on the estimated genetic variance (Figure 6, where smaller groups correspond to more groups, and thus more fixed effects for group).

This pattern does not resemble the pattern of bias with the number of fixed effects and the amount of information per random effect as found by Engel and Buist (1998). First, bias was mostly positive in our results, whereas Breslow and Lin (1995) and Engel and Buist (1998) observed mostly negative bias. Second, the number of fixed effects for group and time increases when groups and observation intervals become smaller. Hence, bias is expected to show a trend towards positive values when groups and observation intervals become smaller. However, the number of groups did not affect the estimate (Figure 6), and bias decreased when moving from intermediate observation intervals to short intervals (Figures 2 and 3). Moreover, the information per random genetic effect increases when the observation interval becomes smaller because more observations are required to cover the epidemic. Based on Engel and Buist (1998), more information per random effect is expected to go together with a trend towards positive bias (or from a negative bias to zero bias). Our results, however, show the opposite trend, where going from intermediate to short observation intervals brings a positive bias down to about zero. Hence, overall we see a different pattern than expected based on knowledge of the bias of variance component estimates from a GLMM.

This difference may reflect that results of Engel and Buist (1998) refer to a classical threshold model, that is, a GLMM with a probit link‐function, whereas our GLMM has a complementary log–log link function. Moreover, our data included a relatively large number of repeated observations on each individual (~75 in the default scenario), so many observations on the same random genetic effect, which may have limited the bias due to the PQL algorithm. The absence of the expected pattern of bias for a GLMM, together with the strong increase in the group*time variance when the observation interval increased, suggests that the bias in our results may be more related to the offset becoming outdated due to new infections and recoveries, than to the use of the PQL algorithm.

In our simulation and analysis, we assumed that the exposure of susceptible individuals to infection is determined by the fraction of infectious individuals in the observation interval. Depending on the specific infection, however, infectious material may accumulate over time in the environment. In that situation, the exposure of susceptible individuals will depend on the infectious fractions in the group in the past and also on the decay of infectious material over time. Statistical methods to estimate infectious disease parameters for such cases have been developed by Chang and de Jong (2023).

Next to susceptibility, other host genetic traits may affect the basic reproduction ratio of an infectious disease and are therefore relevant to selection. Particularly, infectivity, which is the propensity of an animal to infect herd mates once it has become infected itself, and the rate at which it recovers from infection are key traits affecting the basic reproduction rate of an infection. Given that there is evidence for genetic variation in both of these traits (Anacleto et al. 2019; Biemans et al. 2019; Prentice et al. 2022), two questions seem relevant for further investigation: (1) How does genetic variation in infectivity and recovery rate, if not accounted for in the analysis, affect genetic estimates for susceptibility, and (2) can accurate estimates for genetic parameters and breeding values for infectivity and recovery rate be obtained within a GLMM framework.

GLMMs can be used to estimate genetic effects for infectivity (Anche et al. 2015; Biemans et al. 2019). However, previous studies show that estimates for infectivity effects are much more sensitive to experimental design than the estimates for susceptibility (e.g., Anche et al. 2015; Pooley et al. 2022). Nevertheless, the potential of selection on infectivity to reduce the prevalence of infectious diseases in livestock might be even larger than the potential of susceptibility, because genetic variation infectivity has not yet been harvested by breeders (Tsairidou et al. 2018). Thus, there is a need for further research into the suitability of GLMMs to estimate genetic parameters for infectivity and the corresponding data requirements.

Several studies have used GL(M)M to estimate fixed and random effects underlying infectious disease transmission (e.g., Becker 1989; Velthuis et al. 2003; Anche et al. 2015; Biemans et al. 2019). Anacleto et al. (2015) and Pooley et al. (2020, 2025) presented an alternative approach using Bayesian methodology, either for estimating single gene or polygenic host effects. That methodology showed good estimates for susceptibility, infectivity and the duration of the infectious period (Pooley et al. 2025). The GLMM can be implemented in standard software used in animal breeding, such as ASREML (Gilmour et al. 2015), and requires limited computing time. The Bayesian implementation, however, is more flexible as it can handle uncertainties in the data (e.g., regarding the infection status due to poor diagnostic tests), is less affected by infrequent sampling of infection status, and requires fewer assumptions but potentially greater computation time.

Our results indicate that accurate estimation of genetic parameters for susceptibility with GLMM requires frequent sampling of the infection state of individuals. The sampling interval should at least not exceed the average duration of the infectious period, but is preferably much shorter, to prevent bias in the estimates of genetic variance. In practice, achieving such small sampling intervals is labour‐intensive and might be unfeasible, especially in large populations. However, the current rapid developments in automated phenotyping could change this. Investments in such systems seem justifiable, given the large financial and societal impacts of infectious diseases in livestock (Knap and Doeschl‐Wilson 2020).

## Funding

The authors have nothing to report.

## Ethics Statement

The authors have nothing to report.

## Conflicts of Interest

The authors declare no conflicts of interest.

## Supporting information


**Data S1:** jbg70049‐sup‐0001‐supinfo.zip.

## Data Availability

The authors have nothing to report.

## Appendix A Maximum Total Rate

The deterministic SIR model is given by Kermack and McKendrick (1927).

$$\frac{\mathrm{dS}}{\mathrm{dt}}=-\frac{\mathrm{\beta SI}}{N}=-\text{Nβsi}$$

$$\frac{\mathrm{dI}}{\mathrm{dt}}=\frac{\mathrm{\beta SI}}{N}-\mathrm{\alpha I}=N\left(\mathrm{\beta si}-\mathrm{\alpha i}\right)$$

where *N* is the group size, β the transmission rate parameter, α the recovery rate parameter, *S* the number of susceptible individuals, *s* the fraction of susceptible individuals, *s* = *S*/*N*, *I* the number of infected individuals, and *i* the fraction of infected individuals, *i* = *I*/*N*.

To find the maximum total rate, we first find a function relating *i* to *s*, then a function representing the condition for a maximum of the total rate, and finally the maximum TR from the intersection of these two functions.

The relationship between *i* and *s* can be found as follows (Kermack and McKendrick 1927):

$$\frac{\mathrm{di}}{\mathrm{ds}}=\frac{\mathrm{di}}{\mathrm{dt}}/\frac{\mathrm{ds}}{\mathrm{dt}}=-1+\frac{\alpha }{\mathrm{\beta s}}$$

Substituting $\alpha /\beta =1/R_{0}$ and integrating, we find

$$\int \mathrm{di}=\int \left(\frac{1}{R_{0}}\;\frac{1}{s}-1\right)\mathrm{ds}$$

$$i=\frac{1}{R_{0}}\ln\left(s\right)-s+C$$

where $R_{0}$ is the basic reproduction number. The integration constant follows from the start of the outbreak, where *i* → 0 and *s* → 1, giving C=1. Hence, in the deterministic SIR model, *i* is a function of *s*.

$$i\left(s\right)=\frac{1}{R_{0}}\ln\left(s\right)-s+1$$

Next, the total event rate, expressed per individual, is given by

$$\frac{\mathrm{TR}}{N}=\mathrm{\beta si}+\mathrm{\alpha i}$$

The maximum total rate occurs when $d\left(\mathrm{TR}/N\right)/\mathrm{ds}=0$. This is because $d\left(\mathrm{TR}/N\right)/\mathrm{di}$ must be zero at this same point, since *i* is a function of *s* only, as shown above. Hence, we have to solve

$$\frac{d\left(\mathrm{TR}/N\right)}{\mathrm{ds}}=\beta \frac{d\left(\mathrm{si}\right)}{\mathrm{ds}}+\alpha \frac{\mathrm{di}}{\mathrm{ds}}=0$$

Substituting

$$\frac{d\left(\mathrm{si}\right)}{\mathrm{ds}}=s\frac{\mathrm{di}}{\mathrm{ds}}+i$$

Yields

$$\left(\mathrm{\beta s}+\alpha \right)\frac{\mathrm{di}}{\mathrm{ds}}+\mathrm{i\beta}=0$$

Substituting the above expression for di/ds, dividing by β, and substituting $\alpha^{2}/\beta^{2}=1/R_{0}^{2}$ yields

$$\frac{1}{R_{0}^{2}s}-s+i=0$$

Therefore, the condition for the maximum total rate is given by the following relationship between *i* and *s*.

$$i_{\text{maxTR}}=s_{\text{maxTR}}-\frac{1}{s_{\text{maxTR}}\;R_{0}^{2}}$$

The derivation of this condition has ignored the actual relationship between *i* and *s*, that is, it has meaning only when it agrees with a valid combination of *s* and *i*. This point can be found as the intersection of the two functions that relate *i* to *s*, which follows from $i\left(s\right)=i_{\text{maxTR}}$. The result shows that the *s* at which the TR is maximised satisfies the following condition:

$$\frac{1}{R_{0}}\ln\left(s_{\text{maxTR}}\right)+\frac{1}{s_{\text{maxTR}}\;R_{0}^{2}}-2s_{\text{maxTR}}+1=0$$

A close‐form solution for $s_{\text{maxTR}}$ cannot be obtained, but $s_{\text{maxTR}}$ can easily be solved numerically using a root‐finder, such as the uniroot function in the R software (R Core Team 2020). Next, the corresponding *i* follows from the above expression for $i_{\text{maxTR}}$ (or, equivalently, from $i\left(s=s_{\text{maxTR}}\right)$). Finally, the maximum TR per individual, expressed as a function of β and $R_{0}$, equals.

$$\max\left(\frac{\mathrm{TR}}{N}\right)=\beta i_{\text{maxTR}}\left(s_{\text{maxTR}}+\frac{1}{R_{0}}\right)$$

The above condition for $s_{\text{maxTR}}$ shows that $s_{\text{maxTR}}$ and $i_{\text{maxTR}}$ are only a function of $R_{0}$; given the $R_{0}$‐value, they do not depend on *β*. For this reason, at the same $R_{0}$‐value, the maximum TR per individual increases linearly with β.
